# Confirming non-endemicity of podoconiosis in Guatemala and in Idukki District in Kerala (India): a comparison of two approaches potentially suitable for other neglected tropical diseases

**DOI:** 10.1186/s44263-024-00104-y

**Published:** 2024-11-13

**Authors:** Hope Simpson, Mei Trueba, Renata Mendizábal-Cabrera, Sobha George, Chitra Tomy, Silpa T. Sasi, Aran Kartal, Kebede Deribe, Kottarathil Narayanpillai Parameswara Panicker, Gail Davey

**Affiliations:** 1 Brighton and Sussex Medical School, Brighton, UK; 2 London School of Hygiene and Tropical Medicine, London, UK; 3 Center for Health Studies, University of del Valle de Guatemala, Guatemala City, Guatemala; 4 Amrita Institute of Health Sciences, Amrita Vishwa Vidyapeetham, Kochi, Ernakulam India; 5 Children’s Investment Fund Foundation, Addis Ababa, Ethiopia

**Keywords:** Podoconiosis, Case finding, Survey, Spatial targeting, Elimination

## Abstract

**Background:**

Podoconiosis is an underreported lymphoedema whose distribution is uncertain at global level and within endemic countries. Previous work has identified countries with historical evidence of podoconiosis, but which do not currently report cases. Podoconiosis may persist in these countries or have been eliminated due to socioeconomic development. Here we describe two different approaches used to clarify podoconiosis endemicity status in Guatemala and in Idukki District (Kerala State, India).

**Methods:**

Two different epidemiological approaches were used by different research teams, determined by the available resources and contextual factors in the two settings. In Guatemala, where lymphoedema cases are routinely recorded in the health information system, 102 municipalities with suspected cases, historical evidence of podoconiosis, high poverty rates, or environmental suitability for the disease were visited. Active case searches were conducted from July 2016 to October 2018, and suspected cases were clinically examined to confirm or rule out podoconiosis. In Idukki, where lymphoedema cases were not routinely recorded, a population-based prevalence survey for lymphoedema was conducted from September to December 2022, covering 13,664 individuals aged 15 years and older.

**Results:**

Both approaches were effective at clarifying podoconiosis endemicity. In Guatemala, 20 cases with lower limb swelling were investigated. Podoconiosis was ruled out in all cases, and filarial lymphoedema was suspected in three. In Idukki District, 105 cases of lower limb swelling were identified. None was confirmed to have podoconiosis, with post-surgical lymphoedema and hypertension being the most common diagnoses. Active filarial infection was identified in two cases in Idukki District.

**Conclusions:**

These investigations provide evidence that podoconiosis is currently non-endemic in Guatemala and in Idukki District in India. They also demonstrate that population-based surveys and targeted case searches both provide effective ways to explore disease endemicity in areas where this is uncertain. The most appropriate approach depends on a combination of contextual and research-based factors, including evidence for endemicity, resources available, and geographical, population, and health system factors.

## Background

Podoconiosis is a complex non-infectious disease of geochemical origin, leading to chronic lymphoedema and other cutaneous and subcutaneous manifestations affecting the lower limbs. It arises in genetically susceptible populations with barefoot exposure to volcanic soil rich in silicates [1]. Clinically, it presents similarly to lymphoedema caused by lymphatic filariasis (LF), beginning with reversible swelling and progressing slowly to chronic lymphoedema [2, 3]. Advanced stages are characterised by deep folds in the skin, hyperkeratosis, mossy lesions, and joint fixation in severe cases. Like other forms of lymphoedema, including filarial lymphoedema, podoconiosis is associated with acute inflammatory episodes, which become more frequent and severe as the lymphoedema advances, and in turn contribute towards its progression [4]. Case management practices including footcare and hygiene, wound care, exercises and elevation, and treatment of acute attacks are recommended to reduce the incidence of attacks and to limit progression of the lymphoedema [5].

Podoconiosis can be prevented by consistent use of appropriate footwear, and factors such as consistent shoe-wearing and frequent foot washing are protective against the disease [6]. Socioeconomic development and increased shoe-wearing appear to have contributed to the elimination of podoconiosis from northern Africa, generating optimism for its potential elimination from other settings [7]. To support podoconiosis elimination efforts, the Global Atlas of Podoconiosis (GAP) project was initiated in 2017 to clarify the worldwide geographical distribution and epidemiology of the disease, which were uncertain due to a variety of factors [8]. The project’s main aims were to support decisions as to where limited resources available for podoconiosis control should be targeted based on available evidence, and to provide stronger evidence where necessary. Podoconiosis is commonly misdiagnosed as LF, though one key difference is the lack of genital swelling in podoconiosis [3, 9]. Podoconiosis is typically not reported within routine health management systems in endemic countries, and is often not recognised by health workers [10]. One component of the GAP was the systematic compilation of existing data on the disease. This included a systematic literature review [11], which identified records of podoconiosis from 32 countries, including Guatemala and India. This was followed by an evidence consensus study, which used a systematic, quantitative approach to grade the strength of evidence for podoconiosis in every country globally [12].

In Guatemala, certain villages at altitudes between 915 and 1830 m above sea level were reported endemic for a form of lymphoedema in the 1920s and 1930s [13, 14]. The disease was locally described by Rodolfo Robles as *pseudo-leprosy* because of its similarity to Hansen’s disease. Tests on tissue and blood samples did not identify any pathogens, and the investigation concluded that the disease was associated with barefoot exposure to soil [13]. Ernest Price, who first described podoconiosis in a monograph on the disease published in 1990 [15], considered the evidence in Guatemala to be strong enough to recognise ‘pseudo-lepra’ as podoconiosis. Guatemala is considered non-endemic for LF [16, 17].

In India, there is also strong historical evidence for podoconiosis occurrence. This includes surveys conducted between 1974 and 1982 in the districts of Bikaner (Rajasthan State), Aizawal (Mizoram State) and Imphal East and West (Manipur State), all of which were non-endemic for LF. These surveys found rates of lymphoedema between 0.05 and 0.6% in people who had never visited an LF-endemic area [18]. Podoconiosis had also been suspected in the district of Nagpur (Maharashtra State), due to geological similarity to known podoconiosis endemic areas and a high proportion of lymphoedema cases without filarial infection [19]. Additionally, there had been reports of lymphoedema cases of unknown origin in an non-LF-endemic district of Rajasthan [20]. The National Filaria Control Programme (NFCP) of India was launched in 1955 and provided extensive data on the prevalence of lymphoedema and filarial infection through its initial mapping activities [21]. As podoconiosis was not formally described in the medical literature until 1984 [22], it was not considered as an alternative diagnosis in people with lower limb lymphoedema at that time. However, several findings from these surveys point to the possibility of podoconiosis endemicity. Firstly, it was found that the majority of people affected by lymphoedema were negative for filarial infection (before the initiation of mass treatment) [23]. This was consistent with earlier studies, including one in Kerala which found only 12 cases of filarial infection out of 130 lymphoedema cases [24]. Other surveys of hill settlements in Kerala found disease rates higher than the rate of filarial infection, and noted an absence of genital manifestations among people with the disease [25].

Various epidemiological approaches can be used to confirm disease epidemiology within defined geographical areas. Community-based active case searches led to the initial description of podoconiosis in Cameroon [26], while in Rwanda, a population-based nationwide survey provided the first documented evidence of podoconiosis in the country since 1976 [27]. While population-based surveys are required for accurate prevalence estimation, these are costly to implement for low prevalence conditions, and the need to rationalise these and to consider alternative approaches where possible has been raised [28]. Facility-based approaches have been used to explore the epidemiology of other neglected tropical diseases (NTDs) of the skin. For example, in Malawi, sampling and laboratory testing of wounds older than 4 weeks from 161 patients yielded the first ever recorded cases of Buruli ulcer (BU) in the country [29]. A similar approach was taken in Sierra Leone [30], which is not known to be endemic for BU but appears to be environmentally suitable and had previous evidence of possible cases [31, 32]. The study in Sierra Leone did not identify any cases of BU, but the authors noted that this did not exclude the possibility of BU endemicity, as there may have been cases at other health facilities or in communities. While there is no positive diagnostic test for podoconiosis, expert examination of clinically suspect cases in health facilities may provide the opportunity to confirm endemicity, and allows for a higher number of suspect cases to be examined over the same time compared to a community-based approach.

Based on the above-mentioned work, Guatemala was identified as a high priority for confirmatory studies. To initiate the study, the research team contacted researchers based in Guatemala who had previously been involved with onchocerciasis epidemiological evaluations and elimination efforts [33]. The study in India was initiated after researchers from the Amrita Institute of Medical Sciences in Kerala contacted the GAP team to support investigation of podoconiosis endemicity in India. In this article, we describe, compare and contrast two different approaches aiming to confirm contemporary endemicity status of podoconiosis in Guatemala and India. Here we compare the two approaches, first describing their development in each context and the factors influencing this process, and then elaborate upon the methodologies applied. We also reflect on the commonalities and distinctions between the two approaches and their relative advantages and disadvantages, which are somewhat context dependent. In this manner, this paper demonstrates different approaches available to clarify podoconiosis endemicity, highlights considerations that can influence the design of epidemiological evaluations for various diseases and contexts, and provides evidence of podoconiosis endemicity status in Guatemala and in Idukki District.

## Methods

Cross-sectional studies with the aim of confirming podoconiosis endemicity were conducted in Guatemala and Idukki District, India. Different approaches were used in the two settings.

### Guatemala

#### Study team

In Guatemala, the team consisted of one Guatemalan biochemist with extensive experience in NTDs and epidemiological evaluations (RMC), one Spanish-speaking medical anthropologist (MT), and two Guatemalan research assistants (NL and JI) with extensive experience of NTDs and other infectious disease projects with marginalised communities in the study area.

#### Study setting

Guatemala is an upper-middle-income country and the most populous in Central America, with more than 17 million inhabitants [34]. Most of the population is considered Latino (56%), but there are also Mayans (42%), Xincas (2%) and other minority groups. Although the official language is Spanish, 22 Mayan languages are spoken. Guatemala’s economy is diverse, but agriculture continues to be one of its strong components, mainly with products such as coffee, bananas, sugar, and vegetables. The literacy rate is 81.5%, and the average years of study among the population aged 7 years and older is 5.3 in females and 5.8 in males, respectively [35]. As of 2017, 59.3% of the population lived below the national poverty line, with this figure being higher in rural communities [36]. Country-wide, 88% of the population has electricity at their household, 45% has a toilet connected to a drainage network, and 54% cooks with firewood [35]. In Guatemala, health services are free: 70% of health care is covered by the Ministry of Health (MoH), 17.5% by social security, 12% by the private sector, and 0.5% by the army. However, it is estimated that only 48% of the population is covered by MoH and social security services [37].

#### Identification of risk areas

We first established risk areas based on available secondary data representing the locations of historical cases of podoconiosis reported by Robles and Rivera [13, 14], geographical characteristics (elevation between 1000 and 2800 m above sea level) [38], and socioeconomic population-based characteristics (poverty rates of 60% or higher) [39]. ArcGIS 10.8 (Redlands, CA) [40] was used to summarise environmental datasets at municipality level and to produce the map.

Parallel to this, we sought to identify suspected cases via two separate routes. First, we sent a short electronic survey to medical doctors and health professionals working in all health centres in the country. The electronic survey, which was sent together with information on the symptoms, development, and treatment of podoconiosis, asked respondents whether they were familiar with cases presenting with the symptoms of podoconiosis. Second, we identified cases of lymphoedema and related conditions reported through the national health information system from 2014 to 2015. We used data provided by the MoH Sistema de Información Gerencial de Salud (SIGSA). We included all cases classified as follows: other noninfective disorders of lymphatic vessels and lymph nodes (I89); lymphoedema not elsewhere classified (I89.0); other specified noninfective disorders of lymphatic vessels and lymph nodes (I89.8); and noninfective disorder of lymphatic vessels and lymph nodes, unspecified (I89.9) by International Classification of Diseases, Tenth Revision (ICD-10) codes [41].

#### Development of the methodological approach

The approach was designed to be geographically broad in that the team aimed to cover all municipalities which were potentially endemic based on historical evidence of podoconiosis, contemporary evidence of lymphoedema, or environmental and social risk factors for podoconiosis.

Municipalities with a clear overlap of geographical and socioeconomic risk factors and reports of suspected cases were prioritised, but some municipalities without clear geographical risk factors were also visited, considering the potential for cases to have migrated there from other parts of the country.

#### Data collection

Data collection took place between July 2016 and October 2018. Prior to the start of the study, a workshop aiming to raise awareness of podoconiosis and the aims of the study was convened with researchers and stakeholders working on skin disease and NTDs in Guatemala (MT, GD, RMC). Before data collection, the two research assistants (NL and JI) received tailored training covering podoconiosis risk factors, symptoms, differential diagnosis, and recommendations for prevention and treatment. Two to four weeks before visiting the selected municipalities, we contacted the relevant health centres to inform them of the study and to ask them to initiate the community sensitisation process. We provided information about podoconiosis and asked them to display posters illustrating the disease, its symptoms at different stages, and its management. We also requested that they asked patients to refer any possible cases known to them to the centre or research team.

**Fig. 1:**
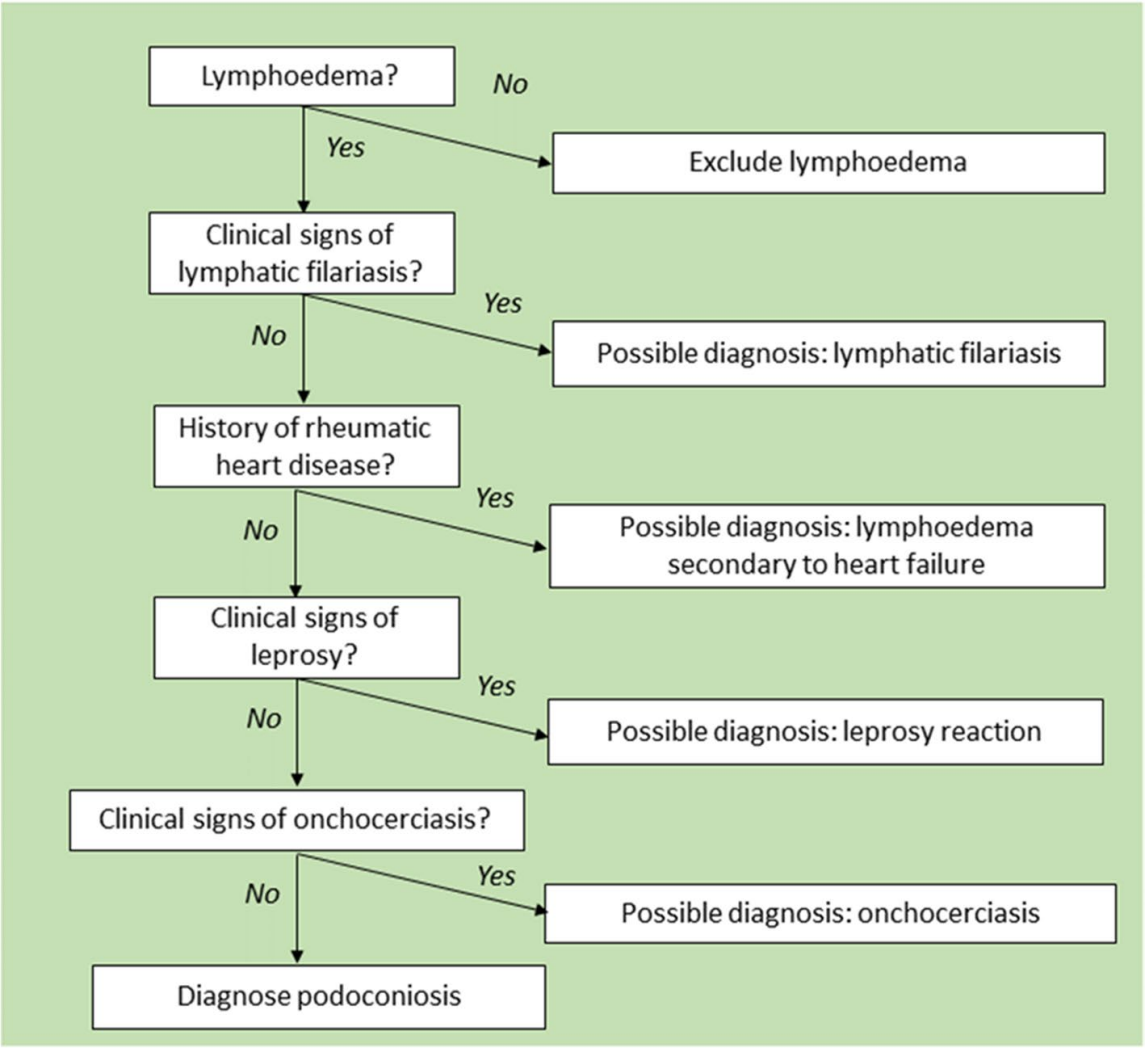
Clinical evaluation of suspect cases of lymphoedema used in Guatemala

The criteria for inclusion of possible cases were that the person suffered from non-specific swelling of the lower limbs (unilateral or bilateral) and was over 18 years of age. All individuals meeting these criteria were examined. We did not set upper or lower limits on the number of possible cases to examine. In accordance with the ethics approval for this project, all cases identified for clinical exploration were provided with information about the project and asked to consent to participate in the study by signing the informed consent document. When health professionals identified possible cases, they arranged for the study team to conduct a clinical examination at the person’s home or in the clinic. Consent was sought by the research team and, if provided, they underwent full clinical evaluation following the algorithm shown in Fig. 1.

In order to broaden the data collection and include possible cases unknown to local health personnel or individuals without access to healthcare, the study team also visited the streets, markets, churches and places of social gatherings in the targeted municipalities, with permission from community leaders. The team showed photographs of podoconiosis cases and asked people about the existence of possible cases of podoconiosis. If someone mentioned a suspected case, we requested their contact information (telephone number, address, or directions) to arrange a visit to their household. We also visited other municipalities which were identified by health workers or community members during these visits. When located, suspected cases were given information about the research and its aims and were asked to provide informed consent. Once all questions about the project were answered and consent was obtained, we first conducted an interview to collect key differential diagnosis information and then performed a clinical evaluation. We also asked suspected cases and their household members if they knew other people who had similar symptoms in order to broaden the data collection. Potential cases identified through this stage underwent the same examination process as suspect cases identified by health professionals. When unable to locate the cases, we provided their name and contact details to local health professionals and rearranged the visit.

The algorithm used for the clinical evaluation of leg swellings observed in Guatemala, adapted from Sime et al. [42], can be seen in Fig. 1.

### India

#### Study team

In India, the team was led by an expert clinician in the field of LF (KNP), who also had experience in diagnosing podoconiosis. The data collection activities were coordinated by three researchers in the field of community medicine. To aid in data collection, seven local health volunteers were trained, with one among them taking on the role of supervisor.

#### Study setting

Idukki is one of fourteen districts in Kerala State, with an estimated population of 1,090,000 in 2022 [43, 44]. It is mountainous and densely forested and generates two thirds of the energy used in Kerala through hydroelectric power [44]. It is sparsely populated, with just 4.7% of the population in urban areas, and agriculture is the most common occupation[44] [45]. According to the 2011 census, the literacy rate was 94.4% in females and 96.7% in males, 99.0% of households have electricity, 82.3% use an improved drinking water source and 97.5.6% use improved sanitation facilities [46]. Of the total population, 1.11% are considered poor [47]; Scheduled Tribes (ST; those officially recognised by the state) make up 5% of the total population [45]. The indigenous groups predominantly reside in the areas of Munnar, Marayur, Mankulam, Adimali and Udumbannur. Of particular significance, Edamalakkudy holds the distinction of being the only tribal panchayath in the state of Kerala with an entirely indigenous population.

The district consists of 54 sub-units (52 grama panchayaths and two municipalities) which are subdivided into wards. As of 2018, there were six hospitals, 41 primary health centres and 13 community health centres, with 10 hospital beds per 10,000 population in the district [47]. The Ministry of Health and Family Welfare of India aims to eliminate LF by 2027, and mass drug administration (MDA) for LF was started in Kerala in 2004, in all districts apart from Idukki, Wayanad and Pathanamthitta, which are non-endemic for LF. In 2018, there was no formal delivery of LF MMDP services given the district’s non-endemic status.

#### Identification of risk areas

In India, risk areas were identified using multiple criteria similar to those used in Guatemala and fully described elsewhere [48]. The 668 districts of India were ranked in terms of priority for investigation based on the incidence of lymphoedema cases known to the health system, environmental suitability for podoconiosis and relative poverty. Additionally, it was assumed that incident cases of podoconiosis in LF-endemic districts could benefit from morbidity management and disability prevention (MMDP) services delivered through the National Vector Borne Diseases Programme (NVBDP), so districts which did not implement these services were prioritised. In total, 35 districts nationwide were ranked high priority for investigation. These districts were spread across 17 states and included an estimated total population of 59.6 million in 2020 [49]. The local research team based at Amrita Institute of Medical Sciences elected to work in Kerala State, as this was most feasible for them to access.

#### Development of the methodological approach

The team elected to focus on a single district, using a cross-sectional design with a population-based survey to estimate the burden of disease. This intensive strategy was deemed necessary because lymphoedema MMDP services were not delivered through the MoH in the study district when the study was implemented, meaning that an approach to identify cases unknown to the health system was required. In addition, this would provide prevalence estimates of lymphoedema and podoconiosis and allow description of the spatial distribution of cases identified. This district represented 0.1% of all (668) districts in India, or 2.9% of the 35 districts considered highest priority for confirmatory mapping surveys. Idukki District was randomly selected from the three districts in Kerala which had been ranked high priority for investigation.

#### Sample size calculation

The required sample size for the survey was estimated at 13,418 using a standard sample size formula. This was based on an expected prevalence of 2.1 per 1000 population (reflecting conservative estimates for the prevalence of lymphoedema) with an absolute precision of 0.001 at a 95% confidence level, assuming a design effect of 1.5 and a participation rate of 90%. The sample size was divided equally across all 54 sub-units of the district, from each of which one ward was selected at random using the lottery technique.

#### Data collection

Seven local health volunteers were selected and trained on research ethics, requesting and recording informed consent, and the data collection methods. The survey was conducted between September and December 2022. In each cluster, data collectors implemented a house-to-house survey, starting at a central point of the community and following random walks until the sample size was reached. At each house in which an adult aged 18 years or older was present, surveyors introduced themselves and sought and recorded written informed consent from all household members aged ≥ 15 years. They showed pictures of podoconiosis symptoms from a flipbook and asked all participants whether they had experienced any swellings consistent with the images, as well as examining them for signs of swelling on the lower limbs. Data were collected on individuals’ age, gender, education, occupation, place of residence, shoe-wearing practices and foot hygiene practices. Household-level data included Water, Sanitation and Hygiene (WASH) variables and socioeconomic status defined by the type of ration card issued to the household by the State Government under the National Food Security Act [50]. Data were collected using the Epicollect5 software package [51] on Android smartphones [52].

**Fig. 2:**
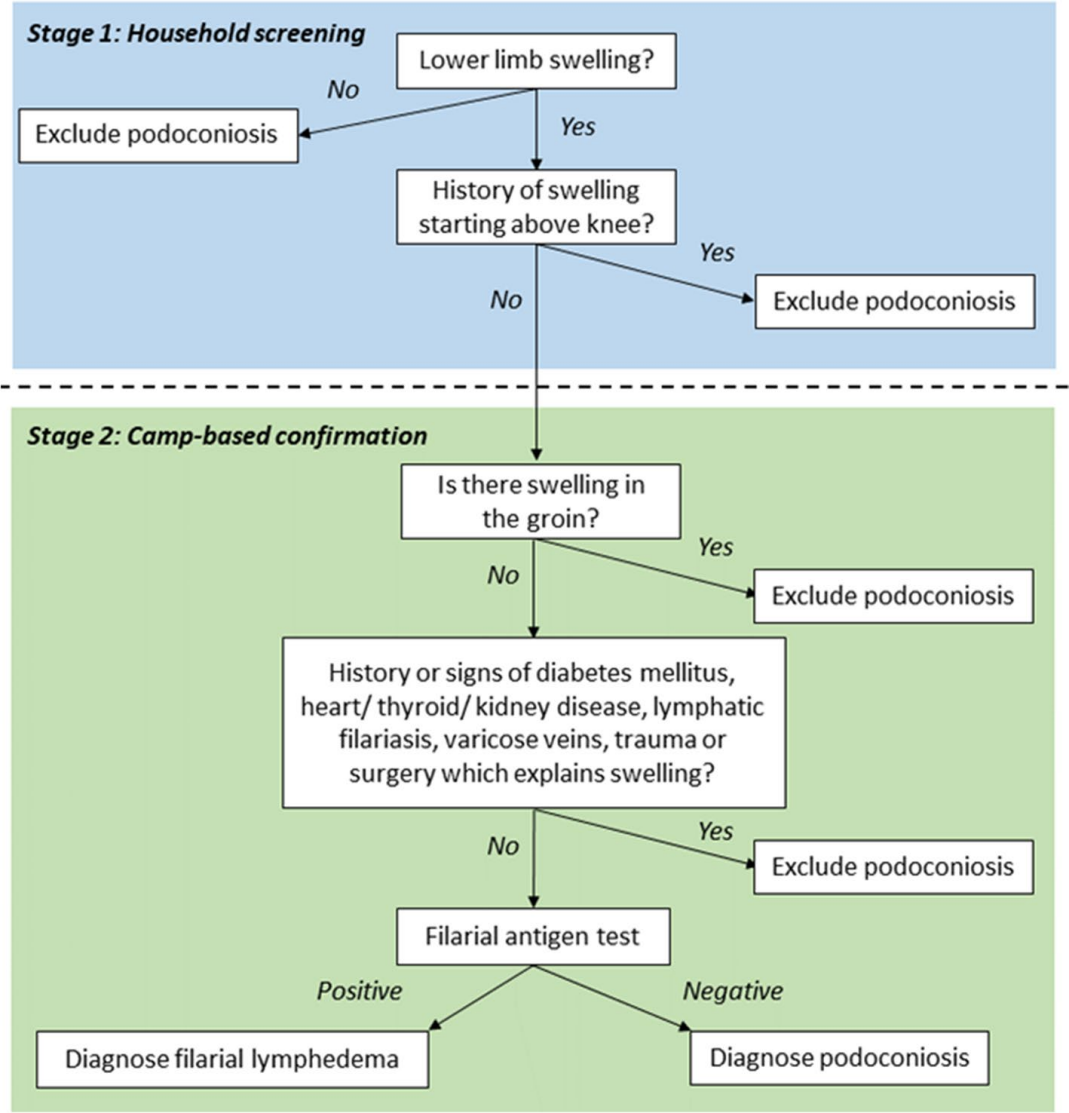
Clinical flowchart used for confirmation of cases of lymphoedema in India

Suspected cases (individuals with any observed or reported sign of swelling on the lower limbs) were invited to a nearby venue for confirmatory examination by medical doctors who were part of the research team (SG, CT and STS). For individuals with lymphoedema, patient histories and clinical indications were used to identify possible differential diagnoses of podoconiosis. This was followed by filarial antigen testing using Bioline Filariasis Test Strips (Alere Abbott), performed according to the manufacturer’s instructions. The clinical process used to confirm the diagnosis of suspect cases in India is represented by the flowchart in Fig. 2.

#### Data analysis

A descriptive analysis was performed to summarise general characteristics of survey participants and confirmed cases. Clopper-Pearson intervals (exact intervals) were used to calculate 95% confidence intervals around estimates of proportions. The map was produced using ArcGIS 10.8 (Redlands, CA) [40].

## Results

### Guatemala

**Fig. 3:**
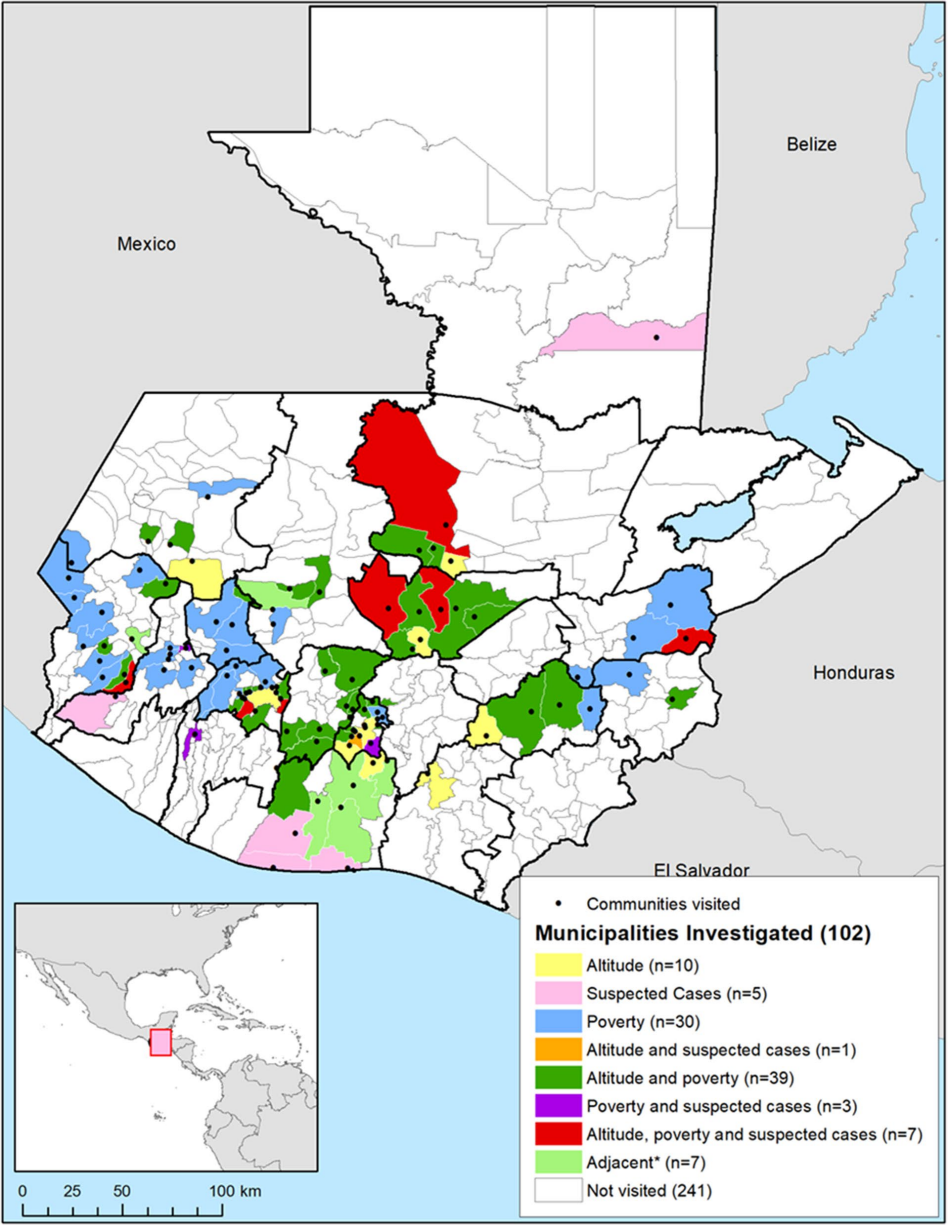
Municipalities and communities of Guatemala visited for podoconiosis active case search activities and reasons for targeted investigation. Country boundaries from the Second Administrative Level Boundaries (SALB) dataset from the United Nations Geographic Information Section [53], Guatemala subnational administrative boundaries from the United Nations Office for the Coordination of Humanitarian Affairs (Guatemala) [54]

Seven municipalities were identified as high priority for investigation, with altitude between 900 and 2000 m above sea level, relatively high poverty, and lymphoedema cases recorded on SIGSA (Fig. 3). Thirty-nine had relatively high poverty and suitable altitude, three had relatively high poverty and known lymphoedema cases, and one had lymphoedema cases and suitable altitude. Thirty were visited due to poverty levels alone, ten due to altitude alone, and five due to lymphoedema notifications alone. Seven municipalities were visited because they were adjacent to municipalities with suspected cases and/or near volcanoes.

In total, we visited 102 municipalities (out of 340 in Guatemala, 30%), located in 16 of the 22 departments in the country, covering 100% of the municipalities in risk areas. The estimated total population within these municipalities in 2016 was 4.34 million, representing 26.7% of the total population.

Figure 3 above shows the location of the visited municipalities in relation to altitude, suspected cases, and the proportion of population living in poverty. The black points signal the visited municipalities.

A total of 20 suspected cases with non-specific lower limb swelling were identified from 15 municipalities located in 10 different departments. There were 8 males and 12 females, with an age range of 23–80 years old (the majority being over 50). Thirteen wore shoes, four had never used footwear, and for three cases we were not able to obtain information on footwear use.

Three cases were examined by a podoconiosis specialist (GD) and were all diagnosed with suspected filarial lymphoedema. Two cases declined to be examined. Photographs of nine other suspected cases were reviewed by the podoconiosis specialist, but podoconiosis was ruled out in all cases. Other possible diagnoses included leg trauma, cardiovascular disease, and cancer. Six cases were reported by health workers and follow-up was attempted. Of these six, five were deceased and one was not traceable. Overall, no cases of podoconiosis were diagnosed. Local health centres and the Pan American Health Organization (PAHO) were notified of the three suspected cases of filarial lymphoedema, for whom filarial antigen testing was recommended.

### India

**Fig. 4:**
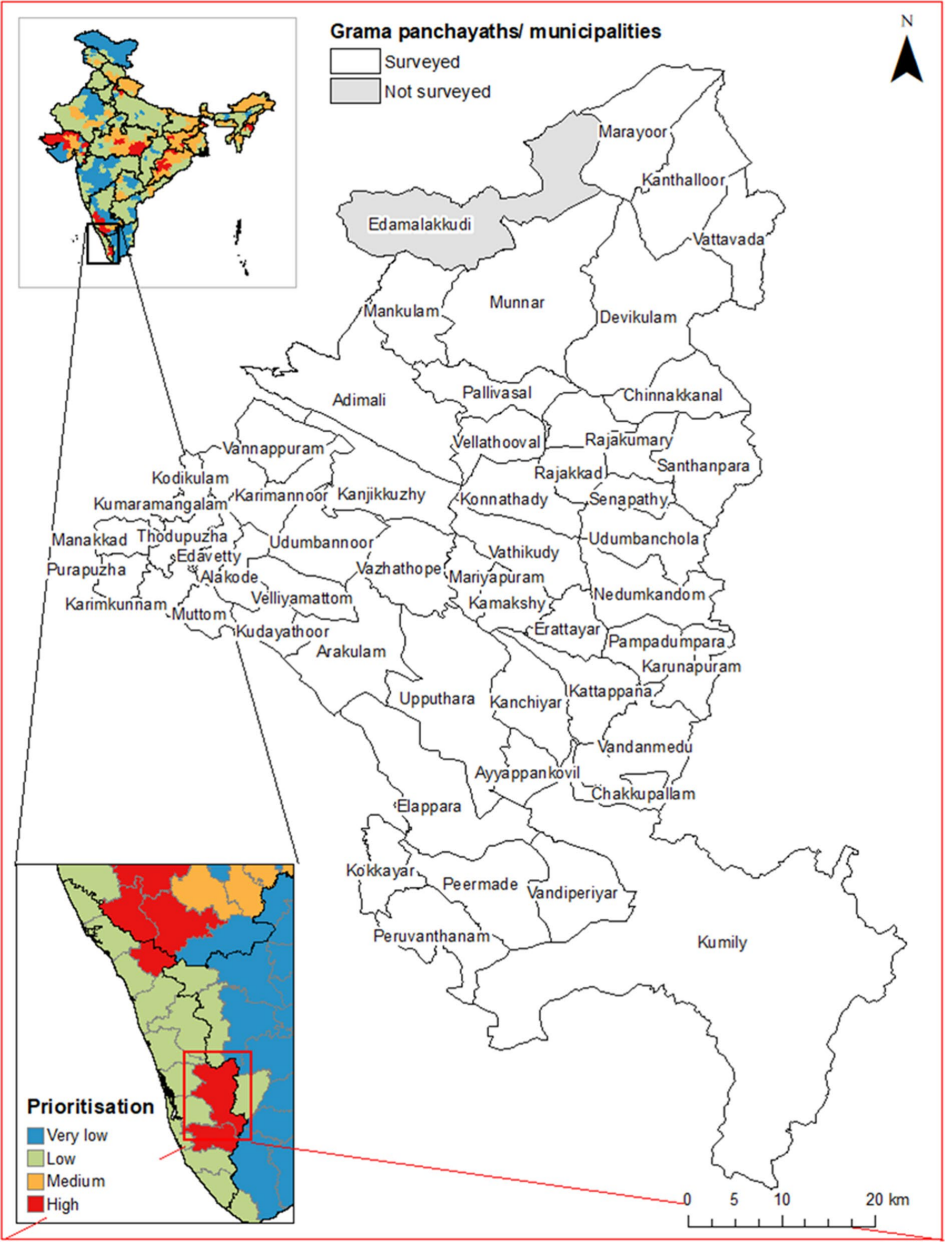
Grama panchayaths and municipalities surveyed in Idukki District, showing the location of the district within India and the district-level prioritisation scores assigned in [48]. State and district boundaries from Geographical Analysis [55], grama panchayath/municipality boundaries from Open Data Kerala [56]

The survey team visited 51/52 panchayaths and the two municipalities in the district (Fig. 4). One panchayath (Edamalakkudy; a tribal hamlet) was not surveyed because the team did not receive authorisation from local authorities.

#### General characteristics of participants

In total, 13,664 individuals were screened. Age of participants ranged from 15 to 99 years with a median of 45 (interquartile range 29–58). Participant demographic characteristics are shown in Table 1. The literacy level was high, with 13,193 participants (96.6%) having attended school or college. According to household ration card possession, the proportion above the poverty line was 46%. Only 447 participants (3.3%) had a soil or cow dung floor inside the house. The mean age of first shoe wearing was 7.5 years. Most participants (11,729; 85.8%) reported washing their legs more than once per day.

**Table 1:** Demographic characteristics of study participants in Idukki District (India)

Category	Number	Proportion (95% CI)
**Gender**		
Male	6868	50.3 (49.4–51.1)
Female	6796	49.7 (48.9–50.6)
**Religion**		
Hindu	7693	56.3 (55.5–57.1)
Christian	5111	37.4 (36.6–38.2)
Muslim	821	6.0 (5.6–6.4)
Others	39	0.3 (0.2–0.4)
**No. of household members**		
≤ 5	10,968	80.3 (79.6–80.9)
> 5	2696	19.7 (19.1–20.4)
**Education level**		
Illiterate	184	1.3 (1.2–1.6)
Literate	287	2.1 (1.9–2.4)
School	9800	71.8 (71–72.5)
College	3393	24.8 (24.1–25.6)
**Occupation**		
Jobless	1017	7.4 (7–7.9)
Student	1794	13.1 (12.6–13.7)
Daily labourer	2211	16.2 (15.6–16.8)
Agriculture	1930	14.1 (13.5–14.7)
Housewife	3825	28 (27.2–28.8)
Private job	2103	15.4 (14.8–16)
Govt. job	212	1.6 (1.4–1.8)
Business	438	3.2 (2.9–3.5)
Retired	134	1.0 (0.8–1.2)
**Socioeconomic status (according to colour of ration card)**		
Above poverty line	6332	46.3 (45.5–47.2)
Below poverty line	7084	51.8 (51–52.7)
Extremely poor	248	1.8 (1.6–2.1)
**Marital status**		
Unmarried	3242	23.7 (23–24.4)
Married	9271	67.8 (67.1–68.6)
Widowed	1074	7.9 (7.4–8.3)
Separated	77	0.6 (0.4–0.7)
**Type of house**		
Kutcha—mud floor, uncemented walls and thatched roof	1393	10.2 (9.7–10.7)
Pucca—cemented floor, cemented walls and cemented or tiled roof	9814	71.8 (71.1–72.6)
Mixed—combination of the above types	2457	18.0 (17.3–18.6)
**Floor of house**		
Improved—tile, cement and/or granite/marble	13,213	54.7 (96.4–97)
Unimproved—soil and/or cow dung	133	57.9 (0.8–1.2)
Mix of improved and unimproved	314	3.1 (2.1–2.6)
Not categorised	4	0.2 (0.0–0.1)
**Water source**		
Piped only	3027	22.2 (21.5–22.9)
Piped + well/borewell	1141	8.4 (7.9–8.8)
Piped + well/borewell and surface	8	0.1 (0.0–0.1)
Piped + surface	222	1.6 (1.4–1.9)
Well/borewell only	8028	58.8 (57.9–59.6)
Well/borewell + surface	342	2.5 (2.2–2.8)
Surface only	896	6.6 (6.1–7.0)

#### Characteristics of lymphoedema cases confirmed by medical team

There were 117 suspected cases with leg swelling identified (Table 2). Of these, 12 were excluded as they reported swelling that started above the knee. The remaining 105 were invited for confirmatory examination and all attended. Lymphoedema was identified in 24 cases, corresponding to a district-level prevalence of 17.6 cases per 10,000 population (95% confidence interval 11.3–26.1 per 10,000). Among these (including some cases with multiple diagnoses) were 9 post-surgical cases, 8 cases of hypertension, 6 cases of diabetes mellitus, 5 with a history of kidney disease, 4 with a history of heart disease, 3 with thyroid disease, 3 with loss of sensation in the legs (indicating possible peripheral neuropathy) and 3 with LF (1 with history and 2 with active filarial infection confirmed by antigen testing). Podoconiosis was ruled out in the study population after full examination.

**Table 2:** Characteristics of lymphoedema cases confirmed by medical team in Idukki District (India)

Characteristics	*n*	% (95% CI)
**Gender**		
Male	11	45.8 (25.6–67.2)
Female	13	54.2 (32.8–74.4)
**Swelling since birth**		
Yes	0	0 (0–14.2)
No	24	100 (85.8–100)
**Bilateral swelling**		
Yes	9	37.5 (18.8–59.4)
No	15	62.5 (40.6–81.2)
**Fungal infection**		
Yes	0	0 (0–14.2)
No	24	100 (85.8–100)
**Swelling in groin**		
Yes	0	0 (0–14.2)
No	24	100 (85.8–100)
**Signs/history of other causes of lymphoedema**		
Present	16	66.7 (44.7–84.4)
Absent	8	33.3 (15.6–55.3)
***Other causes identified (may be multiple)***		
**Post-surgical**		
Yes	9	37.5 (18.8–59.4)
No	15	62.5 (40.6–81.2)
**Hypertension**		
Yes	8	33.3 (0–55.3)
No	16	66.7 (44.7–100)
**Diabetes mellitus**		
Yes	6	25 (0–46.7)
No	18	75 (53.3–100)
**Kidney disease**		
Yes	5	20.8 (7.1–42.2)
No	19	79.2 (57.8–92.9)
**Heart disease**		
Yes	4	16.7 (4.7–37.4)
No	20	83.3 (62.6–95.3)
**Thyroid disease**		
Yes	3	12.5 (2.7–32.4)
No	21	87.5 (67.6–97.3)
**Peripheral neuropathy**		
Yes	3	12.5 (2.7–32.4)
No	21	87.5 (67.6–97.3)
**Lymphatic filariasis**		
Yes	3	12.5 (2.7–32.4)
No	21	87.5 (67.6–97.3)

*CI* Confidence interval (Clopper Pearson)

## Discussion

We conducted epidemiological investigations aiming to confirm the current endemicity status of podoconiosis in Guatemala and Idukki District of India. We used different approaches in the two countries, implementing health facility- and community-based active case searches in Guatemala and a population-based prevalence survey in Idukki District. Neither investigation identified cases of podoconiosis, but lymphoedema due to other causes was identified in both settings. In this section, we discuss the development and implementation of both methodologies and their suitability for future investigations, and evaluate our findings with reference to existing literature before describing the studies’ strengths and limitations.

Guatemala and India were identified as targets for further investigation based on historical reports of possible podoconiosis cases, dating from the 1920s and 1930s in Guatemala [13, 14] and from the 1970s to the early 2000s in India [18, 20]. This study provides strong evidence that podoconiosis is now non-endemic in both settings. Assuming that the disease described as ‘*pseudo-lepra*’ by Robles in Guatemala was really podoconiosis [13], our results would indicate that the disease has been eliminated, probably due to socioeconomic development, as also believed to have occurred in other countries [15]. Alternatively, podoconiosis may never have been endemic in Guatemala. In Idukki District, it is not possible to say whether the disease was ever locally endemic.

With a total population of approximately 16 million in Guatemala and 1.4 billion in India, there was a clear need to target the approach to epidemiological investigation in both countries, given also the uncertainty about the contemporary endemicity of the disease. In both countries, we approached this using a combination of secondary data review and engagement with doctors and public health professionals prior to starting data collection activities. We believe this is a useful and effective way to prepare for exploratory epidemiological investigations when the endemicity of target diseases is unknown, or their prevalence is very uncertain.

In Guatemala, the existence of a single unified health management information system (SIGSA) facilitated the identification of contemporary cases of unclassified lymphoedema and similar conditions known to the health system. In addition, the professional experience and network of the local research team enabled successful engagement of medical doctors across the country via the electronic survey. In India, the team relied on reports of lymphoedema from clinicians and public health practitioners at state level, and in many districts this information was not available. However, in Kerala State, where the research team was based, these secondary data were comprehensive. This highlights how in larger countries such as India, a sub-national approach to planning may be most effective for the planning of targeted activities.

The team in Guatemala took a broad view on indications of possible endemicity and visited all municipalities with historical evidence of podoconiosis cases, environmental risk factors, or high rates of poverty and suspected cases known to the health system. These totalled 102 (30% of all municipalities in Guatemala) with a combined population of 4.34 million. The team used an active case search approach targeting both health facilities and communities, visiting all health facilities and key public areas within targeted municipalities. This was less resource intensive than a population-based survey, enabling a broad geographical reach. Facility-based case searches are a suitable way to explore the potential endemicity of diseases suspected to be under- or mis-diagnosed where cases of the target condition are likely to be able to access services and to be recorded [29, 57, 58].

For the community-based active case searches in Guatemala, the prior experience of the research team in community-based health programmes, their familiarity with the local communities, and the support of the MoH aand of local traditional leaders helped to ensure engagement and participation by the local population and health workers. In design, the community-based component was similar to that of an earlier study on podoconiosis in the northern highlands of Cameroon [26]. The study in Cameroon also included extensive laboratory testing of patient samples and entomological investigations to exclude filariasis as a cause of the pathology identified. These investigations are recommended in countries where LF is known to be endemic.

One limitation of the study in Guatemala was that the team were unable to obtain diagnostic tests to confirm filarial infection. Guatemala is not recognised to be endemic for LF [16], so filarial antigen tests were not planned to be used as part of the diagnostic algorithm. Consequently, cases of lymphoedema resulting from LF infection were suspected but could not be confirmed. Guatemalan health personnel are not trained to collect and examine samples for the parasitological diagnosis of LF and local distributors do not sell rapid or ELISA tests because of the high costs and lack of demand in the country. Unfortunately, we were also unable to obtain tests with international cooperation. Another limitation of the study design was that it was not able to provide estimates of prevalence or the number of people reached. As such, while the study indicates that Guatemala is non-endemic for podoconiosis, we are unable to quantify the uncertainty associated with this finding.

In contrast, a population-based survey was implemented in Idukki District. Population-based surveys are designed to provide unbiased estimates of disease prevalence, which are required to classify the endemicity and to confirm elimination of podoconiosis following implementation of control programmes [7]. In a nationwide population-based podoconiosis survey in Rwanda, 1.3 million individuals were screened through community health workers, and 914 cases were confirmed by expert examination [59]. The survey design also enabled analysis of the spatial distribution of cases and prediction of prevalence at district level and finer scales. A nationwide survey was unfeasible in India, which is more than 100 times bigger than Rwanda by population and area. We targeted a single district which had no formal provision of lymphoedema care through routine health services, assuming that incident cases of podoconiosis in districts which delivered MMDP services for lymphoedema through the NVBDP would benefit from these services even if misdiagnosed, and that districts where MMDP services were not provided should be prioritised. The lack of dedicated lymphoedema services meant that facility-based screening for cases was not possible.

A limitation of the study in India was that the team were denied access to one panchayath inhabited by indigenous groups. Indigenous groups constitute 5.03% of the total population of Idukki District [45]. In Kerala, indigenous populations have been found to have lower levels of access to safe water compared to the general state population, and high rates of walking barefoot outside of the house, suggesting a higher level of risk for podoconiosis [60].

In Guatemala, the identification of possible cases of filarial lymphoedema raises the possibility of LF endemicity in the country, which is currently classified as non-endemic for LF [61]. This may warrant further exploration to confirm whether there is a risk of filarial transmission in the country. There is still uncertainty on the endemicity of podoconiosis in other districts of India, of which thirty-four were considered high priority for surveys based on the same criteria used to target Idukki District [48]. Another five districts had previous evidence of podoconiosis cases [18, 20], but were considered lower priority for surveys for various reasons—one had relatively low rates of poverty and no lymphoedema known to the health system, while four were endemic for LF, suggesting that lymphoedema care services would already be provided through the NVBDP. These districts should be considered for future investigations to explore podoconiosis endemicity status in India, which might be best planned at the state level. Provision of MMDP services for LF morbidity continues to expand in India, with 82 MMDP clinics opened in Kerala since 2018 [62]. As access to care for people with lymphoedema expands, the feasibility of examining known cases for podoconiosis increases. This also relies on cases being recorded within the health information system so that they are traceable. Clinical staff in targeted districts could be given information on the signs of podoconiosis and requested to notify suspect cases. Such facility-based approaches would be much less resource-intensive than surveys, allowing wider implementation with the same financial resources, although they would not provide prevalence data.

Other countries where the current endemicity status of podoconiosis is uncertain include Burundi, Cape Verde, São Tomé and Príncipe and Sudan [12]. In these countries, we suggest that confirmatory epidemiological investigations should be planned with reference to existing provision of care services for lymphoedema, engagement with local health workers and experts in community medicine and public health, and environmental and socioeconomic risk factors. Survey approaches could be made more efficient by specifically targeting communities predicted to be most at risk of podoconiosis, using published estimates of podoconiosis suitability for the African continent for instance [63]. In countries where podoconiosis is considered to have been eliminated, further research to explore the drivers of elimination would provide evidence which may strengthen current elimination efforts in other countries.

## Conclusions

These studies provide strong evidence that podoconiosis is currently non-endemic in Guatemala and Idukki District of India. In a research context, active case searches and population-based surveys offer viable means to explore the potential endemicity of diseases whose incidence is unknown. Active case searches are generally less costly than surveys, allowing a broader geographical reach with the same resources. Facility-based case finding is most effective in settings where cases of the target condition are likely to access routine health services, but may be misdiagnosed. Community-based approaches are necessary to identify cases not known to the health system, and surveys are required for robust prevalence estimation, which can also indicate the need for initiation of dedicated control programmes. For surveys and active case searches, it is crucial to consider whether the sampled population is representative of the population of interest, or whether certain subgroups may have been systematically excluded. A sensitive approach to case finding, led by people trusted by communities and healthcare workers, is essential for population engagement.

## Data Availability

The datasets analysed during the current study are available from Harvard Dataverse: Simpson H, Trueba M, et al. "Replication Data for: Confirming non-endemicity of podoconiosis in Guatemala and in Idukki District in Kerala (India): a comparison of two approaches potentially suitable for other neglected tropical diseases", 10.7910/DVN/JUOWKP, Harvard Dataverse, V1 (see reference [64]).

## Abbreviations

GAP: Global Atlas of Podoconiosis
LF: Lymphatic filariasis
NFCP: National Filaria Control Programme
NTD: Neglected tropical disease
BU: Buruli ulcer
MoH: Ministry of Health
SIGSA: Sistema de Información Gerencial de Salud (Health Management Information System)
ICD-10: International Classification of Diseases, Tenth Revision
MMDP: Morbidity management and disability prevention
ST: Scheduled Tribes
WASH: Water, Sanitation and Hygiene
PAHO: Pan American Health Organization
CI: Confidence interval
